# Effects of Rainfall on *E. coli* Concentrations at Door County, Wisconsin Beaches

**DOI:** 10.1155/2009/876050

**Published:** 2010-02-21

**Authors:** Gregory T. Kleinheinz, Colleen M. McDermott, Sarah Hughes, Amanda Brown

**Affiliations:** ^1^Department of Biology and Microbiology, University of Wisconsin-Oshkosh, 800 Algoma Boulevard, Oshkosh, WI 54901, USA; ^2^Door County Soil & Water Conservation Department, 421 Nebraska Street, P.O. Box 670, Sturgeon Bay, WI 54235, USA

## Abstract

Rainfall and its associated storm water runoff have been associated with transport of many pollutants into beach water. Fecal material, from a variety of animals (humans, pets, livestock, and wildlife), can wash into beach water following rainfall and result in microbial contamination of the beach. Many locales around the world issue pre-emptive beach closures associated with rainfall. This study looked at eight beaches located in Door County, Wisconsin, on Lake Michigan to determine the impact of rainfall on *E. coli* concentrations in beach water. Water samples were collected from beach water and storm water discharge pipes during rainfall events of 5 mm in the previous 24 hours. Six of the eight beaches showed a significant association between rainfall and elevated beach water *E. coli* concentrations. The duration of the impact of rainfall on beach water *E. coli* concentrations was variable (immediate to 12 hours). Amount of rainfall in the days previous to the sampling did not have significant impact on the *E. coli* concentrations measured in beach water. Presence of storm water conveyance pipes adjacent to the beach did not have a uniform impact on beach water *E. coli* concentrations. This study suggests that each beach needs to be examined on its own with regard to rain impacts on *E coli* concentrations in beach water.

## 1. Introduction

Across the nation, closures of recreational swimming beaches due to microbial (fecal) contamination of water have prompted research into the source of elevated microorganism concentrations. Fecal pollution may result from point and nonpoint sources [1, 2]. Point sources such as sewage overflows, agricultural runoff, urban storm water, and streams have been linked to increases in microbial loads to natural bodies of water and swimming beaches [3]. Fecal bacteria identified in water are generally derived from either human and/or animal sources [4] and enter recreational swimming beaches through a variety of methods.

Storm water runoff across impervious surfaces such as roads, roofs, lawns, and construction sites has been identified as the greatest pollution source causing beach closures and advisories [5]. Streets and parking lots are responsible for over 54% of the total runoff volume in residential areas and 80% of the total runoff volume in commercial areas [6]. As storm water flows over these impervious surfaces, the water can pick up a variety of pollutants including oil, grease, nutrients, pesticides, phosphorus, copper, zinc, and fecal bacteria [6, 7]. The fecal bacteria in storm water may be from domestic animals such as cattle, horses, dogs, and cats or wild animals such as deer and waterfowl. Eventually, these contaminated waters reach surface waters, and, if near a swimming beach, can result in elevated bacterial concentrations and increased health risks for swimmers [5].

Heavy rainfall and runoff has been implicated in increases in bacterial contamination at beaches along many coastlines [8, 9]. Along the southern California shoreline, swimming beaches are automatically closed or restricted after a rainfall event greater than 2.5 mm, even without microbiological testing of water [8, 10]. Although storms are fairly infrequent in southern California, rainfall events have precipitated microbial contamination exceedences due to storm water runoff [11]. The increase in bacterial concentrations is associated with almost all storms with rainfall greater than 6 mm and with every storm with rainfall greater than 25 mm. There is little effect on microbial contamination of beaches following storms of less than 2.5 mm [8]. 

Studies of the contamination of recreational waters by storm water and nonpoint source runoff have been conducted in a few locations along highly urbanized coastlines [8, 10]. While this research has been focused on urban marine shorelines, the contamination of rural and semiurban freshwater swimming beaches by nonpoint source runoff has not been extensively studied. At several urban beaches in southeastern Wisconsin, the beaches are automatically closed due to increased microbial concentrations in water following a rain event and increased storm water runoff [12, 13]. Data on microbial loading of swimming beaches due to rainfall and runoff have been collected at several Lake Michigan and Lake Superior beaches in Wisconsin [12–14]; however, data quantifying the microbial loads during rainfall and storm water runoff events have not be studied at the mainly rural beaches located in Door County, Wisconsin.

**Figure 1:**
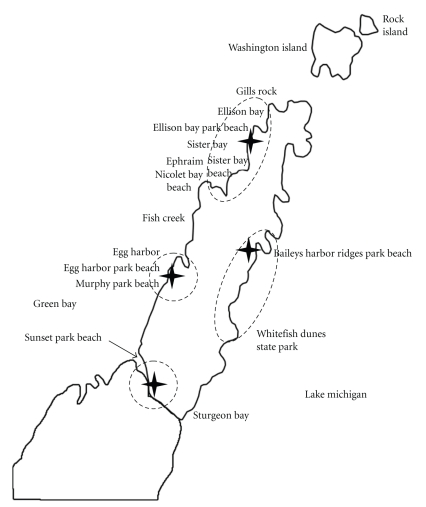
Beaches utilized in this study were located along the Lake Michigan and Green Bay coastlines in Door County, Wisconsin. Stars indicate location of the rain gauges and the dashed lines show the beaches that were studied based on the rainfall at those locations.

Door County is a 75-mile long peninsula located in northeastern Wisconsin. The county is bordered by Lake Michigan to the east and the Bay of Green Bay to the west and has over 300 miles of shoreline with over 30 swimming beaches. Door County is one of the greatest tourist destinations in the Midwest, with more than two million visitors per year. Because many of these tourists visit the beaches in Door County, a minimum of 31 beaches along both sides of the Door County peninsula, at Washington Island, within the Sturgeon Bay Canal, and at three inland lakes (Figure 1) have been sampled for fecal bacteria on a regular basis during the summer swimming season (June 1–August 31), since 2003. In addition, daily rainfall data were collected at multiple locations on the peninsula using rain gauges. Rain gauges were spaced throughout the county to account for differences in meteorological affects from the northern end of the county to the southern end and to provide data for more than one beach (Figure 1).

While the beaches in Door County have been monitored for fecal bacteria, there is little information on the extent rainfall that impacts the water quality of these beaches. Due to the lack of available data related to rainfall impacts on rural beaches, eight beaches were monitored for *E. coli* concentrations following rain events greater than 5 mm (Figure 1). To quantify the microbial loads at these beaches during rainfall and runoff events for comparison to microbial loads during regular beach conditions, the beaches and nearby outfalls (stream, pipe, or urban runoff areas) were monitored at regular intervals (hourly for four hours, at eight hours, at 12 hours, and 24 hours) within one hour of the rainfall event. The overall objective of this project was to determine what impact rainfall had on the *E. coli* concentrations at the selected beaches.

## 2. Materials and Methods

### 2.1. Water Sample Collection

Recreational water samples were collected from the eight beaches (Ellison Bay, Sister Bay, Nicolet Bay, Egg Harbor, Murphy Park, Baileys Harbor, Whitefish Dunes State Park, and Sunset Park) (Figure 1) from water with a depth of 24–30 inches (and approximately 12 inches below the surface) as specified by the requirements of the WI BEACH Act sampling program [15]. All beaches were sampled four days per week for the summer season (approximately Memorial Day to Labor Day). 

Several of the beaches in this study were considered semirural. That is, they were located in small villages (population 250–1000 individuals). These include Egg Harbor, Ellison Bay, Nicolet Bay, and Sister Bay. Several beaches were rural (not in a town or village proper) including Bailey's Harbor, Murphy Park, and Whitefish Dunes State Park. One location, Sunset Park, was in a residential/industrial area (located in a town of approximately 10,000 people). All water samples were collected into 100mL sterile, polystyrene collection bottles (IDEXX Corp., Portland, ME) and placed at 4°C until *E. coli* concentration analysis was conducted. Care was taken to collect samples in a uniform and nonintrusive way as not to contaminate samples with excess disruption of sediments or floating debris. Samples were analyzed within 4 hours of collection. A Wisconsin State Certified Laboratory with a Quality Assurance plan on file with the WI Department of Agriculture, Trade and Consumer Protection (DATCP) maintained by the University of Wisconsin-Oshkosh was utilized for all analysis. Positive, negative, and proficiency testing controls were prepared in accordance with the laboratory's quality assurance plan.

### 2.2. Water Sample Collection after Rainfall Event

To capture rain data near the recreational beaches in Door County, four rain gauges (Crytical Services, Model FT 501, Little Chute, WI) were placed at strategic locations (Ellison Bay, Sturgeon Bay, Egg Harbor, and Bailey's Harbor) throughout the county (Figure 1). Proper placement was critical to ensure that rain sensor readings were an accurate representation of the actual rain measurement rates and amounts that have fallen. Each rain gauge collected rain data for one to three beaches depending on proximity to the gauge. The gauges logged data 24 hours a day, seven days a week throughout the swimming season. The rain gauges reported rainfall amounts to a real-time data logging system. After the data logger system recorded over 5 mm of water accumulation within a rolling 24-hour period, the data system automatically sent notification of the rain event to a designated cell phone number (regardless of the day of week, or time of day). Upon receiving this notification call the sampling described below would commence in less than half an hour from the accumulation of 5 mm of rain in the 24-hour period. 

Following a rain event notification, beach water samples were collected every hour for four hours, then at eight, 12, and 24 hours after the rain fall notification (total of seven samples per beach following the rain event). Beach rain event samples were collected from water with a depth of 24 inches in the center of the beach following standard sample collection protocol. Outfall (pipe or stream) water samples were collected during the first three hours of the rain event (total of three samples per outfall) at locations that contained these features. Amount of outfall discharge was not determined. All rain event water samples were placed at 4°C until *E. coli* concentrations were determined. All beaches, except Nicolet Bay Beach, had ten post rainfall sampling events. Due to logistical and access issues Nicolet Bay Beach had four events. If rainfall was sufficient to trigger multiple rain event notifications in one 24 hour period, then multiple sampling events were conducted.

### 2.3. Sample Analysis

The defined substrate test, Colilert (IDEXX Corp., Portland, ME), was used to analyze all samples for *E. coli *concentration [16]. Incubation and microbial enumeration from samples were conducted following the manufacturer's recommendations. All results were reported as most probable number (MPN) of *E. coli* per 100 mL of water.

### 2.4. Statistical and Graphical Analysis

Seasonal mean *E. coli* concentrations were calculated for each beach by averaging all *E. coli* concentrations determined for routine monitoring to satisfy the requirements of the BEACH Act (four samples/week, regardless of rainfall). Mean *E. coli* concentrations during rain events were calculated by averaging *E. coli* concentrations from samples collected following rain event notifications (i.e., seven samples/rain event notification, see above). Statistical analysis (ANOVA and Scheffe Matrices) was performed with Systat 11.0. Figures were generated with Microsoft Excel 2004.

## 3. Results

During the summer of 2007 each of the selected beaches, except Nicolet Bay Beach, had ten sampling events conducted. Nicolet Bay had four collections. The impact of rainfall on *E. coli* concentrations in beach water was not uniform. Some locations, such as Nicolet Bay, Whitefish Dunes State Park Beach, and Egg Harbor Beach, showed no significant positive relationship between rainfall and beach water *E. coli* concentrations. Other beaches such as Sister Bay Beach, Ellison Bay Beach, Murphy Park Beach, and Bailey's Harbor Beach showed time limited impacts from the rainfall with elevated concentrations of *E. coli* in the beach water. Last, some locations, such as Sunset Park Beach, showed a lesser impact of rainfall on beach *E. coli* concentrations, with peak *E. coli* concentrations occurring immediately after a rainfall.

Bailey's Harbor Beach and Murphy Park Beach showed a significant increase in *E. coli* concentration due to rainfall (*P* < .001 and.001, resp., Table 1). When the post-rainfall sample intervals were separated, however, it became apparent that the significant impact was only present in the first four hours after the rainfall (Tables 2 and 5). By the time the eight hour samples were collected, the difference was no longer significant (*P* = .094 and.189, resp.), and the significance decreased at 12 hours (*P* = .320 and.3) and at 24 hours (*P* = .899 and.977). At Bailey's Harbor Beach, the outfall pipe adjacent to this beach showed a significant difference between storm water outfall concentrations of *E. coli* and seasonal beach *E. coli* concentrations (*P* = .001). *E. coli* concentrations from this outfall pipe for the first three hours of sampling after a rainfall event showed a strong correlation with each other (*r* ^2^ = 0.864). This indicates that the storm water *E. coli* discharging to Bailey's Harbor beach water is sustained for several hours. *E. coli* concentrations measured in beach water samples collected during rainfall events, however, do not show good correlations with the *E. coli* concentrations discharging from the storm water pipe (*r* ^2^ = −0.268).

The amount of rainfall that triggered the sampling event was not correlated with *E. coli* concentrations in beach water for either Bailey's Harbor or Murphy Park Beaches. Rainfall sampling events were triggered by an accumulation of 5 mm of rain in the previous 24-hour period. *E. coli* concentrations in beach water at these two beaches did not show significantly greater increases if rainfall in the previous days was substantially greater than 5 mm. 

In contrast to Bailey's Harbor and Murphy Park Beaches, *E. coli* concentration from Egg Harbor Beach, Nicolet Bay, and Whitefish Dunes State Park Beach was not significantly positively impacted (*P* = .849,.041, and.110, resp., See Tables 1, 3, 6, and 9) by rain events. At Egg Harbor Beach there is one outfall pipe adjacent to the beach and *E. coli* concentrations measured for the first three hours of storm water discharge do not correlate well with *E. coli* concentrations found in beach water (*r* ^2^ = 0.345 at the three hour sampling point) at the same times (Table 3). All outfall concentrations of *E. coli* were significantly different from those of the seasonal beach water average (*P* = .001); however, this discharge does not appear to show up in the beach water itself (*P* = .849). 

There are no outfalls near Nicolet Bay or Whitefish Dunes State Park Beaches and they are the most rural of all locations included in this study. Whitefish Dunes is characterized by a large sandy beach area, natural dunes, and the largest avian population present on the beach within Door County, WI [17]. Nicolet Bay beach is located within Peninsula State Park and is surrounded by a large wooded park acreage.

Ellison Bay Beach and Sister Bay Beach also showed a significant increase in *E. coli* concentration due to rainfall (*P* = .001 for both beaches; Tables 1, 4, and 7). When the postrainfall sample intervals were separated, it became apparent that the significant impact was present in the first 12 hours after the rainfall (*P* = .002 and.024, resp.). By the time the 24-hour samples were collected the difference was no longer significant (*P* = .772 and.754). There is no storm water pipe located near Ellison Bay Beach itself, but there is a large amount of impervious surface adjacent to the beach and a large avian population. 

Sister Bay Beach, with two storm water outfall pipes located adjacent to the beach, is perhaps the most complicated beach in this study. The two storm water outfall pipes adjacent to this beach each showed a significant difference between storm water outfall and seasonal beach *E. coli* concentrations (*P* < .001) (Table 7). Outfall pipe #1 (located closest to the beach proper) had a constant concentration of *E. coli* discharging to surface water during the first three hours of a rain event, and these outfall pipe *E. coli* concentrations were significantly different from the beach *E. coli *concentration means (*P* < .001 at 1 and 2 hours, and.024at 3 hours). Pipe #2 (located on the same side of the beach as pipe #1, but more distant from the beach) *E. coli* concentrations were significantly different from *E. coli* concentrations in beach water at 1 hour (*P* = .001) and 2 hours (*P* = .003) but was not significantly different at 3 hours (*P* = .259). This indicates that the storm water *E. coli* discharge from pipe #1 to the beach is fairly sustained, or at least remains at consistently elevated concentrations of *E. coli* for three hours beyond a rain event. Discharge from pipe #2 is affected by “first flush” and shows significantly elevated *E.coli* discharge for only the first two hours after a rain event. Pipe #1's *E. coli* concentrations correlated well with the *E. coli* concentrations from beach water samples collected during the same sampling event (*r* ^2^ = 0.920), while pipe #2's *E. coli* concentrations do not correlate well with *E. coli* concentrations measured during the same time-frame (*r* ^2^ = −0.582). 

At Sister Bay Beach there is no correlation between the amount of rainfall prior to the sampling event and the concentration of *E. coli* in beach water. Any amount of rainfall in the days prior to the sampling event had a similar effect on beach *E. coli* concentrations. At Ellison Bay Beach, however, there were good positive correlations between the amount of rainfall in the days prior to the sampling event and the concentration of *E. coli* measured in beach water at the time of sampling (e.g., *r* ^2^ = 0.78 for beach water *E. coli* at 3 hours postrainfall event and amount of rainfall in previous 24 hours; *r* ^2^ = 0.83 for beach water *E. coli* at 3 hours postrainfall and amount of rainfall in previous 48 hours, etc.). It appears that there is a direct connection between overland runoff from impervious surfaces at this beach and beach water *E. coli* concentrations, with more rain resulting in more runoff and greater beach *E. coli* concentrations. 

Sunset Park Beach also showed a significant increase in *E. coli* concentration due to rainfall (*P* = .011; Table 1). When the postrainfall sample intervals were separated, it became apparent that the significant impact must be immediate (occurring in a time frame of less than 1 hour postrainfall event) and was not as great as that observed at other locations (Table 8). The Sheffe tests revealed no significant differences between *E. coli *at the postrainfall sampling times and the seasonal mean of *E. coli* at this beach. There are no storm water pipes located near the beach, but there is a large park adjacent, with a large number of waterfowl present.

## 4. Discussion

Effects of rainfall on beach water *E. coli* concentrations were not uniform, even at beaches within one county, all located on Lake Michigan. Of the eight beaches we sampled for this study, four different beach “profiles” emerged with respect to rainfall and *E. coli* measurements in beach water. Bailey's Harbor and Murphy Park Beaches (profile 1) showed an elevation of *E. coli* concentration in beach water with rainfall, but the effect was seen only in the first three hours of rain event sampling (Tables 2 and 5).

It is plausible that “first flush” (from stormwater discharge pipe or from overland flow) during the rain event caused the increase in *E. coli* observed at the beach and currents and dilution were able to alleviate this input within eight hours after a rainfall event. These beaches are located on and open to large bodies of water (Lake Michigan and Bay of Green Bay, resp.), facilitating dilution of *E. coli* runoff.

Egg Harbor, Nicolet Bay, and Whitefish Dunes State Park Beaches (profile two), on the other hand, did not show a positive association between rainfall and elevated *E. coli* concentrations in beach water (Table 1). Nicolet Bay (Peninsula State Park) and Whitefish Dunes State Park Beach were the most isolated and remote location in the study. It seems logical that lack of large human or domestic/agricultural animal populations, as is the case at Nicolet Bay and Whitefish Dunes, would result in production of less fecal material and a reduced chance of beach contamination by storm water runoff or outfall conveyance systems during rainfall events. The other location, Egg Harbor Beach, showed unexpected results however, due to its location within a small village and its close proximity to other more impacted sites (Murphy Park Beach and Sister Bay Beach). Additionally, the steep grade at Egg Harbor Beach, high overland flow, and apparent commonalities with many other Door County beaches would have made Egg Harbor a likely candidate for adverse rain impacts on beach water quality. This lack of impact could be due to long-shore currents or dilution at the site of storm water discharge.

The third observed beach profile occurred at Ellison Bay and Sister Bay Beaches (profile 3) and was characterized by a significant association between rainfall and beach water *E. coli* concentrations, but for a longer time frame than was seen in profile 1 (Tables 4 and 7). At these two beaches, rainfall had a significant impact on beach water *E. coli* concentrations for approximately twelve hours following the rainfall event trigger. It again is plausible that first flow during the rain events caused the increase in *E. coli* observed at the beach and the currents and dilution were able to alleviate this input within 12–24 hours after a rainfall event

Sunset Park Beach represents the fourth beach profile identified in this study (profile 4). While *E. coli* concentrations during a rainfall event and the seasonal mean *E. coli* concentration in water were significantly different (*P* = .011) (Table 1), the impact must have been immediate (minutes following the start of the rainfall), as examination of the *E. coli* concentrations at rain event sampling times (beginning one hour after the event was triggered and 5 mm of rainfall had already accumulated) was not significantly different from the seasonal mean *E. coli* concentration from beach water (Table 8). Again, it is plausible that overland flow during the rain event caused the increase in *E. coli* observed at the beaches and that currents and dilution were able to alleviate this input without a long-term impact on *E. coli* concentrations at the beach. Additionally, the Sheffe test is a bit more sensitive than the general ANOVA and would be more detailed in explaining specific impacts of rainfall events.

Overall, six of the eight locations studied showed significant impacts on the beach water *E. coli* concentrations as a result of rain events greater than 5 mm within a 24-hour period. The results of this study clearly demonstrate that several beaches within Door County, WI, are impacted by rain events. The significance and duration of this adverse impact, however, is highly beach specific. The results of this study suggest that each beach needs to be examined on its own with regard to rain impacts on *E. coli* concentrations in beach water. The methodologies described in this paper were novel with respect to rainfall-beach interactions and were effective in determining the impact of rainfall on specific locations as well as on the duration that the impact existed for a specific location. These rainfall-beach interaction data are important for beach managers to collect and assess as they develop pre-emptive beach closures related to rainfall and then must justify these closures to their constituents and tourists who frequent the beaches.

## Figures and Tables

**Table 1:** Overall impact of rainfall on *E. coli* concentrations in beach water. Mean *E. coli* concentration in beach water during a rainfall event was compared with the seasonal mean *E. coli* concentration at the same beach by analysis of variance. All beaches, except Nicolet Bay Beach, had a total of ten rain events with 7 samples collected per 24-hour sampling event. Nicolet Bay Beach had four rain sampling events. Bolded *P*-values indicated significantly higher *E. coli* concentrations during rain events (*P* < .05).

Beach name	Mean *E. coli* during rain event	Seasonal *E. coli* mean	Significant difference
	(MPN/100 mL)	(MPN/100 mL)	(*P* value)
Baileys Harbor	225.4	53.4	**.000**
Egg Harbor	41.0	61.4	.849
Ellison Bay	447.7	43.3	**.000**
Nicolet	19.4	26.4	.041
Murphy Park	191.0	115.9	**.000**
Sister Bay	778.3	276.0	**.000**
Sunset Park	187.5	102.1	**.011**
Whitefish Dunes	39.8	48.3	.110

**Table 2:** *E. coli* concentrations (*MPN/100 mL) from beach water collected at time intervals following the triggering of a rain event at Bailey's Harbor beach. Using Scheffe matrix analysis, only *E. coli* concentrations measured in the first four hours after the rain event was triggered were significantly different from the seasonal mean *E. coli* concentration for this beach (*P* < .001). If rainfall was sufficient to trigger multiple rain event notifications on one day, then multiple events were sampled.

Date of Rain Event	Outfall Pipe 1–3 Hours	1–4 hours After	8 hours After	12 hours After	24 hours After
	After Rain Event	Rain Event	Rain Event	Rain Event	Rain Event
05/19/07	1506.6	998.1	13.2	9.8	1.0
05/24/07	690.6	17.3	8.6	17.3	12.2
06/02/07	2084.3	4.1	5.2	4.1	1.0
06/04/07	>2419.6	10.9	56.5	10.9	34.9
06/04/07	n/a	67.0	84.2	67.0	1.0
06/07/07	1908.9	115.3	70.3	115.3	5.3
06/18/07	>2419.6	218.7	248.1	218.7	5.2
06/18/07	n/a	143.9	218.7	143.9	7.4
07/10/07	n/a	22.8	86.2	22.8	1.0
08/20/07	771.7	290.9	816.4	290.9	>2419.6
08/28/07	22.0	28.8	34.1	28.8	2.0

*Most probable number.

**Table 3:** *E. coli* concentrations (*MPN/100 mL) from beach water collected at time intervals following the triggering of a rain event at Egg Harbor beach. There was no significant different between overall *E. coli* concentrations during a rain event and the seasonal mean *E. coli* concentrations for this beach using ANOVA (Table 1). Likewise, using Scheffe matrix analysis, there was no significant difference between *E. coli* concentrations at any sampling times after a rain event and the seasonal *E. coli* mean. If rainfall was sufficient to trigger multiple rain event notifications on one day, then multiple events were sampled.

Date of Rain Event	Outfall Pipe 1–3 Hours	1–4 hours After	8 hours After	12 hours After	24 hours After
	After Rain Event	Rain Event	Rain Event	Rain Event	Rain Event
5/19/07	n/a	35.9	1.0	35.9	11.0
5/24/07	n/a	18.7	12.2	18.7	2.0
6/2/07	1806.2	27.5	118.7	27.5	275.5
6/4/07	60.0	34.5	49.5	34.5	27.9
6/18/07	511.8	4.1	1.0	4.1	1.0
6/18/07	n/a	7.4	1.0	7.4	1.0
6/18/07	n/a	4.1	2.0	4.1	2.0
7/10/07	n/a	18.9	66.3	18.9	3.1
7/11/07	n/a	2.0	2.0	2.0	3.0
7/12/07	n/a	7.4	11.0	7.4	2.0
7/26/207	n/a	12.2	4.1	12.2	1413.6
8/20/07	n/a	122.3	3.1	122.3	12.1

*Most probable number.

**Table 4:** * E. coli* concentrations (*MPN/100 mL) from beach water collected at time intervals following the triggering of a rain event at Ellison Bay beach. Using Scheffe matrix analysis, *E. coli* concentrations measured in the first four hours after the rain event was triggered were significantly different from the seasonal mean *E. coli* concentration for this beach (*P* < .001). Likewise, *E. coli* concentrations measured at 8 hours or 12 hours after the rain event was triggered were significantly different from the seasonal mean *E. coli* concentration for this beach (*P* < .001 and *P* < .002, resp.). If rainfall was sufficient to trigger multiple rain event notifications on one day, then multiple events were sampled.

Date of Rain Event	Outfall Pipe 1–3 Hours	1–4 hours After	8 hours After	12 hours After	24 hours After
	After Rain Event	Rain Event	Rain Event	Rain Event	Rain Event
6/4/07	n/a	1348.9	>2419.6	488.4	613.1
6/4/07	n/a	1783.3	>2419.6	1732.9	461.1
6/4/07	n/a	2202.9	1413.6	1046.2	59.1
6/18/04	n/a	58.1	29.2	41.3	9.7
6/18/07	n/a	41.8	30.5	20.5	13.2
6/20/07	n/a	131.1	55.6	58.3	2.0
7/10/07	n/a	4.3	30.9	29.4	5.2
7/12/07	n/a	29.9	81.6	36.8	4.1
8/20/07	n/a	37.5	48.0	60.9	6.3
8/23/07	n/a	10.3	47.3	37.9	15.6
8/28/07	n/a	114.8	44.3	55.6	5.2

*Most probable number.

**Table 5:** *E. coli* concentrations (*MPN/100 mL) from beach water collected at time intervals following the triggering of a rain event at Murphy Park beach. Using Scheffe matrix analysis, only *E. coli* concentrations measured in the first four hours after the rain event was triggered were significantly different from the seasonal mean *E. coli* concentration for this beach (*P* < .001). If rainfall was sufficient to trigger multiple rain event notifications on one day, then multiple events were sampled.

Date of Rain Event	Outfall Pipe 1–3 Hours	1–4 hours After	8 hours After	12 hours After	24 hours After
	After Rain Event	Rain Event	Rain Event	Rain Event	Rain Event
5/19/07	n/a	1.0	1.0	1.0	69.7
5/24/07	n/a	69.1	79.8	69.1	9.7
6/2/07	n/a	3.1	5.2	3.1	4.1
6/4/07	n/a	98.4	45.0	98.4	1.0
6/18/07	n/a	172.3	344.8	172.3	43.7
6/18/07	n/a	228.2	224.7	228.2	48.0
6/18/07	n/a	248.9	204.6	248.9	43.5
7/10/07	n/a	18.7	54.6	18.7	16.1
7/11/07	n/a	15.8	8.6	15.8	48.0
7/12/07	n/a	16.1	27.5	16.1	6.3
7/26/07	n/a	30.5	24.1	30.5	9.7
8/20/07	n/a	48.1	111.2	48.1	2.0

*Most probable number.

**Table 6:** *E. coli* concentrations (*MPN/100 mL) from beach water collected at time intervals following the triggering of a rain event at Nicolet Bay beach. Overall *E. coli* concentrations during a rain event were not significantly higher than the seasonal mean *E. coli* concentrations for this beach using ANOVA (Table 1). Likewise, using Scheffe matrix analysis, there was no significant difference between *E. coli* concentrations at any sampling times after a rain event and the seasonal *E. coli* mean.

Date of Rain Event	Outfall Pipe 1–3 Hours	1–4 hours After	8 hours After	12 hours After	24 hours After
	After Rain Event	Rain Event	Rain Event	Rain Event	Rain Event
6/20/07	n/a	23.8	8.6	16.0	1.0
7/12/07	n/a	1.0	5.2	22.6	7.4
8/23/07	n/a	16.5	78.5	9.8	40.2
8/28/07	n/a	15.8	7.4	115.3	2.0

*Most probable number.

**Table 7:** *E. coli* concentrations (*MPN/100 mL) from beach water collected at time intervals following the triggering of a rain event at Sister Bay beach. Using Scheffe matrix analysis, *E. coli* concentrations measured in the first four hours after the rain event was triggered were significantly different from the seasonal mean *E. coli* concentration for this beach (*P* < .001). Likewise, *E. coli* concentrations measured at 8 hours or 12 hours after the rain event was triggered were significantly different from the seasonal mean *E. coli* concentration for this beach (*P* < .001 and *P* < .024, resp.). If rainfall was sufficient to trigger multiple rain event notifications on one day, then multiple events were sampled.

Date of Rain	Outfall Pipe #1 1–3 Hours	Outfall Pipe #2 1–3 Hours	1–4 hours After	8 hours After	12 hours After	24 hours After
Event	After Rain Event	After Rain Event	Rain Event	Rain Event	Rain Event	Rain Event
6/4/07	1795.4	392.3	>2419.6	>2419.6	101.4	32.3
6/4/07	n/a	200.4	>2419.6	249.5	95.9	25.4
6/4/07	n/a	n/a	299.8	76.2	160.7	30.5
6/18/07	>2419.6	n/a	1265.1	1046.2	235.9	133.3
6/18/07	n/a	n/a	648.0	325.5	178.5	107.6
6/20/07	2406.6	n/a	2094.7	90.6	101.2	13.5
7/10/07	1664.0	n/a	504.7	114.5	65.0	101.2
7/12/07	657.0	n/a	94.3	26.9	41.9	19.9
8/20/07	1779.3	>2419.6	294.3	48.0	1.0	1.0
8/23/07	n/a	0.0	248.6	73.3	155.3	27.5
8/28/07	>2419.6	>2419	>2419.6	1986.3	980.4	32.3

*Most probable number.

**Table 8:** * E. coli* concentrations (*MPN/100 mL) from beach water collected at time intervals following the triggering of a rain event at Sunset Park beach. While there was a significant difference between the mean *E. coli* concentration during a rain event and the seasonal mean *E. coli* concentrations for this beach using ANOVA (Table 1), Scheffe matrix analysis showed no significant difference between *E. coli* concentrations at each sampling times after a rain event and the seasonal *E. coli* mean.. If rainfall was sufficient to trigger multiple rain event notifications on one day, then multiple events were sampled.

Date of Rain Event	Outfall Pipe 1–3 Hours	1–4 hours After	8 hours After	12 hours After	24 hours After
	After Rain Event	Rain Event	Rain Event	Rain Event	Rain Event
6/4/07	n/a	739.8	238.2	198.9	44.3
6/17/07	n/a	482.7	111.2	68.9	13.1
6/18/07	n/a	39.5	73.8	46.8	18.5
6/18/07	n/a	52.7	46.4	159.7	29.5
6/20/07	n/a	45.6	36.9	187.2	21.3
7/10/07	n/a	594.8	39.3	43.5	4.1
7/11/07	n/a	48.8	13.5	18.7	7.4
7/12/07	n/a	504.1	17.1	13.2	7.4
7/26/07	n/a	182.6	178.5	172.5	42.5
8/28/07	n/a	93.7	45.9	65	25.3

*Most probable number.

**Table 9:** * E. coli* concentrations (*MPN/100 mL) from beach water collected at time intervals following the triggering of a rain event at Whitefish Dunes State Park beach. There was no significant different between overall *E. coli* concentrations during a rain event and the seasonal mean *E. coli* concentrations for this beach using ANOVA (Table 1). Likewise, using Scheffe matrix analysis, there was no significant difference between *E. coli* concentrations at any sampling times after a rain event and the seasonal *E. coli* mean. If rainfall was sufficient to trigger multiple rain event notifications on one day, then multiple events were sampled.

Date of Rain Event	Outfall Pipe 1–3 Hours	1–4 hours After	8 hours After	12 hours After	24 hours After
	After Rain Event	Rain Event	Rain Event	Rain Event	Rain Event
5/19/07	n/a	2.0	1.0	2.0	2.0
5/24/07	n/a	2.0	1.0	2.0	1.0
6/2/07	n/a	1.0	2.0	1.0	2.0
6/4/07	n/a	4.1	4.1	4.1	49.6
6/4/07	n/a	35.9	2.0	35.9	1.0
6/7/07	n/a	4.1	32.7	4.1	1.0
6/18/07	n/a	45.2	37.4	45.2	3.1
6/18/07	n/a	18.9	47.9	18.9	1.0
6/18/07	n/a	9.8	29.2	9.8	9.8
7/10/07	n/a	9.8	21.6	9.8	1.0
8/20/07	n/a	11.8	64.4	11.8	28.8
8/29/07	n/a	3.1	31.3	3.1	9.8

*Most probable number.
